# Enhancing the Flavor Profile of Summer Green Tea via Fermentation with *Aspergillus niger* RAF106

**DOI:** 10.3390/foods12183420

**Published:** 2023-09-14

**Authors:** Minyu Cai, Liyan Huang, Sashuang Dong, Nanxin Diao, Weilian Ye, Zhiye Peng, Xiang Fang

**Affiliations:** College of Food Science, South China Agricultural University, Guangzhou 510642, China; 15625595153@163.com (M.C.); lvy_088@163.com (L.H.); dongsashuang@126.com (S.D.); 15989880586@163.com (N.D.); m19124308171@163.com (W.Y.); hesxjlg0629@163.com (Z.P.)

**Keywords:** summer green tea, *Aspergillus niger*, fermentation, flavor

## Abstract

Summer green tea (SGT) has a low cost and high annual yield, but its utilization rate is limited due to suboptimal quality. The aim of this study is to enhance the flavor of SGT using fermentation with *A. niger* RAF106 while examining changes in its metabolites during this process. The results revealed an elevation in the content of alcohol, alkanes, and nitroxides in tea leaves following the process of fermentation. The predominant volatile compounds identified in tea leaves after undergoing a 6-day fermentation period were linalool, (Z)-α, α, 5-trimethyl-5-vinyltetrahydrofuran-2-methanol, (E)-linalool oxide (furan type), linalool oxide (pyran type), and theapyrrole. These compounds exhibited significant increases of 31.48%, 230.43%, 225.12%, 70.71%, and 521.62%, respectively, compared to the non-fermented control group (CK). The content of non-ester catechins, soluble sugars, and total flavonoids reached their peak on the 4th day of fermentation, exhibiting significant increases of 114.8%, 95.59%, and 54.70%, respectively. The content of gallic acid and free amino acids reached their peak on the 6th day of fermentation, exhibiting significant increases of 3775% and 18.18%, respectively. However, the content of ester catechin decreased by 81.23%, while caffeine decreased by 7.46%. The content of lactic acid, acetic acid, and citric acid in tea after fermentation was 421.03%, 203.13%, and 544.39% higher than before fermentation, respectively. The present study offers a fresh approach for the advancement of SGT.

## 1. Introduction

In China, green tea is classified based on the seasons it is grown in: spring, summer, and autumn. It goes through a process of fixing, rolling, and drying without fermentation [1]. The production of SGT accounts for approximately half of total annual tea production; however, it exhibits a more pronounced bitter and astringent flavor compared to spring green tea [2]. The presence of this characteristic imposes limitations on the processing and utilization of SGT, resulting in substantial resource inefficiency. Catechins, including ester catechins and non-ester catechins, are the main bitter and astringent substances in green tea. The ester catechins, including (-)-epigallocatechin gallate (ECG) and (-)-epicatechin gallate (EGCG), exhibit the highest content, constituting approximately 75% of the total catechin content. Notably, SGT demonstrates the elevated content of ester catechins in comparison to spring tea [3,4]. Ester catechins exhibit a higher degree of astringency and bitterness compared to non-ester catechins. Additionally, there is a positive correlation between the ratio of non-ester catechins/ester catechins and the sensory evaluation score of the tea broth flavor [5,6].

Microbial fermentation is a conventional technique in which the combined action of microorganisms, increased temperature, and optimal moisture content triggers a series of biochemical reactions involving decomposition, oxidation, consolidation, structural alteration, methylation, and glycosylation. As a result, this complex process gives rise to unique flavors and bioactive compounds [7]. Microbial fermented tea exhibits distinct organoleptic characteristics, including a vibrant infusion, unique aroma, natural sweetness, velvety taste, minimal bitterness, and astringency compared to non-microbially fermented tea [8]. The effects of fermentation can vary depending on the microbiota, including bacteria, yeast, and mold. In recent years, there has been a significant research focus on the bacterial fermentation of tea, particularly in relation to kombucha. The process of fermentation leads to changes in the sugar and acid content, resulting in a unique flavor profile. Additionally, the production of aromatic compounds enhances the aroma of the tea [9]. However, tea polyphenols have an inhibitory effect on the growth and reproduction of bacteria, resulting in the relatively prolonged fermentation period of bacteria, which lasts between 1 and 2 weeks. Yeast has often been used to ferment Pu-erh tea, which leads to an increase in tea polyphenols, theaflavins, and catechins while causing a decrease in amino acids, caffeine, flavonoids, thearubigin, and theaflavin [10]. *A. niger* possesses a rich enzyme system, which is not inhibited by tea polyphenols. The fermentation process generates an enzyme system that triggers a cascade reaction, which can lead to the production of volatile and non-volatile metabolites that enhance the flavor and functional properties of tea. Metagenomics has recently facilitated the discovery of *A. niger*’s glycoside hydrolase (GH) genes, which play a pivotal role in pile fermentation and aroma production in dark tea [11]. After the fermentation of steamed green tea by *A. niger* PW-2, the content of catechins significantly decreases, while the content of volatile compounds, including linalool, (-)-α-terpineol, and geraniol, also decrease significantly [12]. Therefore, the different strains of *A. niger* and variations in raw materials result in diverse fermentation outcomes.

In our previous investigation, *A. niger* RAF106, isolated from Pu-erh tea, bioconverted ester catechins of green tea polyphenols in the aqueous solution to non-ester catechins and gallic acid, improving the bioavailability of tea polyphenols and their free radical scavenging activity in vitro was comparable to that of the control group [13]. Additionally, SGT fermented with *A. niger* RAF106, resulting in a significant increase in the content of free amino acids, the elevated content of gallic acid, and an abundance of non-ester catechins. Furthermore, the fermented tea broth exhibited fragrant, mellow, and sweet aftertaste and moderate acidity. In this study, volatile and non-volatile substances in tea were quantitatively analyzed by HS-SPME-GC-MS, electronic nose, and HPLC after the solid-state fermentation of STG with *A. niger* RAF106 as the only inoculant fungus. The purpose of this study is to elucidate the effect of the RAF106 fermentation process of *A. niger* RAF106 on SGT flavor characteristics and lay a foundation for the development and application of high-value SGT.

## 2. Materials and Methods

### 2.1. Schematic Overview of the Experimental Program

**Figure 1:**
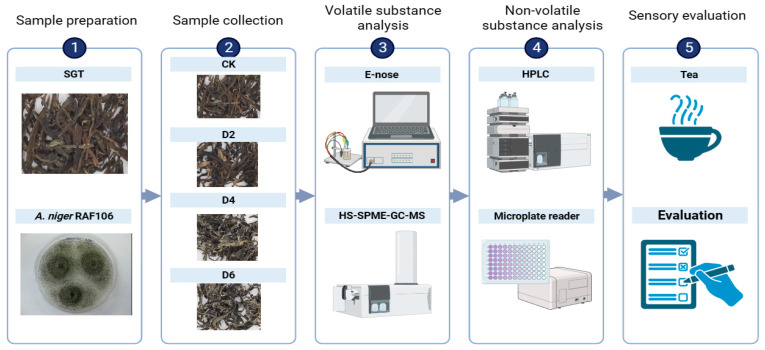
Schematic overview of the experimental program.

This figure illustrates the steps and specific methods of this experiment (Figure 1). In brief, the experiment involved subjecting SGT, inoculated with *A. niger* RAF106, to solid-state fermentation at a temperature of 42 °C for a duration of six days. The tea collected at 0, 2, 4, and 6 days of fermentation were, respectively, designated as CK, D2, D4, and D6. After being dried at 80 °C, volatile and non-volatile substances as well as the sensory differences of the samples (CK, D2, D4, and D6), were measured to analyze metabolic changes occurring in tea during fermentation and ascertain whether *A. niger* RAF106 could enhance the flavor profile of SGT. The aim was to offer a streamlined and efficient approach to tackle the problem of the underutilization of SGT. The performed experiments were consistent with the standard procedures prescribed by the College of Food Science, South China Agricultural University, where the work was performed.

### 2.2. Chemicals

The unspecified chemical reagents were of analytical grade. Foline-phenol was obtained from Solepol Co., Ltd. (Beijing, China). Hydrated ninhydrin stannous chloride, disodium hydrogen phosphate, and anthrone were acquired from Shanghai McLean Biochemical Science and Technology Co., Ltd. (Shanghai, China). Disodium hydrogen phosphate, sodium dihydrogen phosphate, and sodium carbonate were purchased from Guangzhou Chemical Reagent Factory (Guangzhou, China). Anthrone was procured from Shanghai McLean Biochemical Science and Technology Co., Ltd. (Shanghai, China). Ammonium acetate was sourced as chromatographically pure from Tianjin Komeo Chemical Reagent Co., Ltd. (Tianjin, China). Acetonitrile, tetrahydrofuran, trifluoroacetic acid, and dimethyl sulfoxide s chromatographically pure were obtained from Shanghai McLean Biochemical Technology Co., Ltd. (Shanghai, China). Gallic acid (GA), (+)-catechin (C), and (-)-epigallocatechin (EGC) were standardized products purchased from Shanghai Amperexperiment Technology Co., Ltd. (Shanghai, China). (-)-Epicatechin (EC), (-)-epicatechin gallate (ECG), (-)-epigallocatechin gallate (EGCG), Quinicacid and citricacidwere acquired from Aladdin Reagent (Shanghai) Co., Ltd. (Shanghai, China). Concentrated sulfuric acid and ammonium acetate served as standard products acquired from Guangzhou Chemical Reagent Factory (Guangzhou, China). Oxalic acid, malic acid, lacticacid, fumaricacid, and caffeine were standard products bought at Tanmo Quality Assurance Technology Co. (Jiangshu, China). Oxalic acid, malic acid, lactic acid, fumaric acid, and caffeine were standard products bought at Tanmo Quality Assurance Technology Co. (Changzhou, China).

### 2.3. Preparation of Tea Samples

SGT is produced in Yunnan; *A. niger* RAF106 is preserved by the Fermentation Engineering Group, School of Food, South China Agricultural University.

*A. niger* RAF106 suspension: *A. niger* RAF106 glycerol tubes were taken and stored at −20 °C using an inoculation needle to transfer them onto a PDA plate. They were incubated at 30 for 5–7 days. When colonies grew on the surface of the medium, we rinsed them off with sterile water containing 0.1% Tween 80 and transferred them into a 10 mL centrifuge tube. These were shaken well to obtain an evenly distributed suspension of *A. niger* RAF106 clasps. The concentration of the *A. niger* RAF106 suspension was adjusted to 1~2 × 10^8^ per milliliter using a hemocytometer plate.

Preparation of fermented tea: The tea in the fermentation bag was weighed at 100 g and rehydrated with sterile water until it reached a moisture content of 45%. The suspension of *A. niger* RAF106 was uniformly inoculated into the tea leaves at a ratio of 1 mL: 100 g. The inoculated tea leaves are placed in a fermentation incubator at 42 °C for fermentation, with three replicates per group. During the fermentation period, the tea leaves were turned daily, and samples were taken every two days for six days in total. After fermentation, the samples were dried in an oven at 80 °C and then stored in vacuum packaging. The non-fermented tea and teas fermented for 2, 4, and 6 days were designated as CK, D2, D4, and D6, respectively.

### 2.4. Volatile Substance Extraction and Analysis

#### 2.4.1. E-Nose Analysis

Aroma collection method: We weighed 1.0 g of the samples (CK, D2, D4, and D6) separately into 50 mL conical flasks. Each flask was sealed with a plastic wrap they were allowed to stand for 30 min. Subsequently, the aroma detector head of an electronic nose was inserted into each conical flask to collect aroma data.

Detection conditions: The injection flow rate was 300 mL/min, and the cleaning channel required 120 s. Automatic zeroing took 10 s, the sample preparation time was 5 s, and the measurement time lasted for 110 s. Data analysis was performed within the response signal interval of 106~108 s. PEN3 electronic nose comprised a total of 10 sensors. Among them, W1C (S1) detected aromatic components and benzene; W5S (S2) exhibited a high sensitivity to nitrogen oxides; W3C (S3) showed sensitivity toward aromatic components and ammonia; W6S (S4) primarily selected hydrides; W5C (S5) detected alkane aromatic components; W1S (S6) responded to methyls; W1W (S7) reacted to sulfides; W2S (S8) sensed alcohol, aldehydes, and ketones; W2W (S9) detected aromatic components and organosulfides; finally, W3S (S10) demonstrated sensitivity toward long-chain alkanes.

#### 2.4.2. HS-SPME-GC-MS Analysis

Aroma components in the samples were extracted using headspace solid phase microextraction (HS-SPME). Samples (CK, D2, D4, and D6) weighing 1.0 g were placed in a 15 mL extraction bottle and sealed with a lid. Subsequently, the bottle was equilibrated in a 90 °C water bath for 10 min. Afterward, the extraction head consisting of DVB/CAR/PDMS with particle sizes of 30/50 μm was inserted into the extraction bottle for headspace extraction lasting for 30 min. Following this step, it was introduced into the gas chromatography (GC) injection port and desorbed at 250 °C for 5 min before being analyzed by GC-MS.

GC-MS analysis conditions: GC conditions included an inlet temperature of 250 °C and a chromatographic column of TG-5MS (60 m × 0.25 mm, 0.5 μm). The injection mode was set to the splitless mode. The carrier gas used was helium, with a flow rate of 1.2 mL/min. The heating program consisted of maintaining the temperature at 60 °C for 1 min, followed by an increase of 3 °C/min until it reached 150 °C and was maintained for 2.0 min; then the temperature increased at a rate of 3 °C/min to reach 180 °C were it was maintained for another 3.0 min; finally, the temperature was increased to 240 °C at 4 °C/min and kept constant for 2.0 min. MS conditions involved setting the ion source temperature to 230 °C and interface temperature to 230 °C with scanning performed in the range of 35–600 m/z. The volatile substances in the sample were analyzed using a total ion flow diagram (Figure S2).

### 2.5. Non-Volatile Extraction and Analysis

#### 2.5.1. Quantitative Analysis of Catechins and Gallic Acid

The catechin and gallic acid contents were quantified using a Waters 2489 HPLC system (Alliance E2695 High-Performance Liquid Chromatography: Waters Ltd., Milford, MA, USA). Sample extraction was performed in accordance with the China National Institute of Standardization (CNIS) GB/T 8313-2018 [14]. In brief, 0.2 g of tea powder was combined with 5 mL of 70% methanol and subjected to heating in a water bath at 70 °C for 10 min. Subsequently, the mixture was centrifuged at a speed of 3500 r/min for 10 min, and this process was repeated twice. Prior to determination, tea infusion was filtered through a 0.22 μm Zinten filter. The chromatographic conditions were as follows: a Waters XBridge^®^ C18 high-performance liquid chromatography column (250 × 4.6 mm, 5 μm) was used. The mobile phase was prepared in accordance with the specifications outlined (Table S1). The HPLC measurement procedure included the following parameters: column temperature was set at 25 °C, detection wavelength at 280 nm, and flow rate at 1 mL/min. The mobile phase composition was as follows: 0–5 min, 100% A; 5–25 min, 100%-65% A; 25–30 min, 0% A; 30–35 min, 100% A. The retention time of catechins and gallic acid was determined by analyzing the chromatogram (Figure S1). The content of catechins and gallic acid was determined by calculating them using the standard curve (Table S2).

#### 2.5.2. Quantitative Analysis Organic Acid

The organic acid content was quantified using the Waters 2489 HPLC system. The extraction method referred to the China National Institute of Standardization (CNIS) GB/T 8314-2013 [15]. The tea powder (0.3 g) was mixed with boiling water (45 mL) and subjected to extraction in a 100 °C water bath for 45 min with intermittent shaking every 10 min. Following cooling, the solution was further diluted with water to achieve a final volume of 50 mL. Prior to determination, the tea infusion was filtered through a 0.22 μm Zinten filter. The chromatographic conditions were as follows: Waters XBridge^®^ C18 high-performance liquid chromatography column (250 × 4.6 mm, 5 μm), and the HPLC assay determination procedure was as follows: the mobile phase consisted of 0.1% phosphoric acid (A) and methanol (B); the column temperature was set at 27.5 °C; detection wavelength at 210 nm; and flow rate at 0.7 mL/min. The mobile phase composition was as follows: 0–15 min, 99–80% A and 15–20 min, 99% A. The retention time of organic acids was determined by analyzing the chromatogram (Figure S1). Their content was calculated based on the standard curve of organic acids (Table S1).

#### 2.5.3. Analysis of Tea Polyphenols

The polyphenol content in tea was determined using the forintol method, as described by China National Institute of Standardization (CNIS) GB/T 8313-2018 [14]. The method for sample extraction was consistent with the description provided in Section 2.5.1. After the extraction process, we combined 1 mL of the supernatant with a resorcinol reagent (10% concentration, 5 mL) and allowed it to undergo a reaction for five minutes before introducing a Na_2_CO_3_ solution (4 mL, with a concentration of 7.5%). The mixture was diluted to a volume with water, shaken thoroughly, and incubated at room temperature for one hour prior to measuring absorbance using a 96-well plate at a wavelength of 765 nm. The content was quantified based on the standard curve of total polyphenols (y = 0.0074x + 0.0035, R^2^ = 0.9989).

#### 2.5.4. Analysis of Total Free Amino Acids

Referring to the China National Institute of Standardization (CNIS) GB/T 8314-2013 [15], the ninhydrin colorimetric method was used for determination. The extraction procedure employed was identical to that described in Section 2.5.2. A volume of 1 mL from tea infusion was combined with a pH 8.0 phosphate buffer (0.5 mL) and a solution containing 2% ninhydrin solution (0.5 mL). The mixture was heated in a boiling water bath for 15 min and then allowed to cool before adding enough water to reach a final volume of 25 mL. After standing undisturbed for 10 min, the absorbance was measured at a wavelength of 570 nm using a 96-well plate. The content was finally calculated using the standard curve of free amino acids (y = 1.0171x − 0.109, R^2^ = 0.9957).

#### 2.5.5. Analysis of General Flavone

Sample extract preparation involved weighing 0.10 g of the ground tea sample in a 10 mL centrifuge tube, adding 5 mL of 60% ethanol, performing ultrasonication-assisted extraction for 30 min, centrifuging at 8000 r/m for 5 min, collecting the supernatant, repeating the extraction once, and combining the supernatant in a 10 mL volumetric flask for volume adjustment. For measurement purposes, 100 μL of the standard test solution and sample solution was pipetted into a 1.5 mL centrifugal tube. Then, 150 μL of a 5% AlCl3 solution was added, followed by the addition of 100 μL of an acetic acid–sodium acetate buffer (pH = 5.5). The total volume was adjusted to 1 mL with 60% ethanol and left to stand for 30 min. The absorbance value of the extracted solution was measured at 400 nm using spectrophotometry. Finally, the content of the extracted solution was determined based on the standard curve of the total flavonoids (y = 0.0014x + 0.0001, R^2^ = 0.999).

#### 2.5.6. Quantitative Analysis of Caffeine

The caffeine content was quantified using a Waters 2489 HPLC system. The extraction method was the same as in Section 2.5.1. Prior to determination, the tea infusion was filtered through a Zinten filter with a pore size of 0.22 μm. The chromatographic conditions were as follows: Waters XBridge^®^ C18 high-performance liquid chromatography column (250 × 4.6 mm, 5 μm). The HPLC assay determination procedure was as follows: 0.4% phosphoric acid solution (A) and methanol (B) were used for isocratic elution with a ratio of A:B = 75:25. The detection wavelength was set at 280 nm, the flow rate was 0.8 mL/min, the column temperature was maintained at 30 °C, and the injection volume was 10 μL. The content was calculated using the standard curve of caffeine (y = 30,716x + 266,033, R^2^ = 0.9994).

#### 2.5.7. Analysis of Soluble Sugars via Ultraviolet Spectrophotometry

Tea extracts were prepared according to the method described in the China National Institute of Standardization (CNIS) GB/T 8314-2013 [15] for extraction. The quantitative analysis of soluble sugars was conducted using the anthrone reagent [16]. The extraction method was the same as in Section 2.5.2. In 1.5 mL centrifugal tubes, 100 μL of a glucose standard assay solution and the sample extract were separately taken, with tertiary water used as a blank control. In an ice-water bath, 900 μL of anthrone–sulfuric acid solution was slowly added and shaken well. The mixture was then heated in a boiling water bath for 9 min, cooled, and left for 30 min before measuring the absorbance at 625 nm. The content was calculated based on the standard curve of soluble sugar (y = 0.0078x + 0.0421, R^2^ = 0.9996).

### 2.6. Sensory Evaluation

The sensory assessment team consisted of five experts who possessed professional qualifications, and their selection was determined by evaluating their consistent and reliable performance. Before the sensory assessment, they all signed a paper version of the Sensory Informed Consent form. Sensory evaluation was based on three factors, including color, aroma, and taste, according to the China National Institute of Standardization (CNIS) GB/T 23776-2018 [17], with slight modifications. The assessment process involved infusing three grams of samples in 150 mL of water at a temperature of 85 °C for a duration of three minutes. Subsequently, color, aroma, and taste were promptly assessed. According to the scoring criteria (Table S3), the total score is as follows:

Total score = 20% (a) + 30% (b) + 50% (c)(1)

The components were as follows: (a) color, (b) aroma, and (c) taste.

### 2.7. Data Analysis

Each experiment was repeated three times, and the experimental data were expressed as the mean ± standard deviation (Mean ± SD). Data were recorded and calculated using MS Office form tools. GraphPad Prism 9 was used to plot and analyze the significance (ANOVA) of the data. WinMuster (1.6.2.22) software was used to generate the PCA and loading plots of the E-nose data. HS-SPME-GC-MS analytical data were analyzed using Wiley/NIST spectral library matching to characterize unknown compounds, with the peak area normalization method employed for the relative quantification of each compound.

## 3. Results

### 3.1. Changes of Tea Volatiles during Fermentation

#### 3.1.1. E-Nose Evaluation

**Figure 2:**
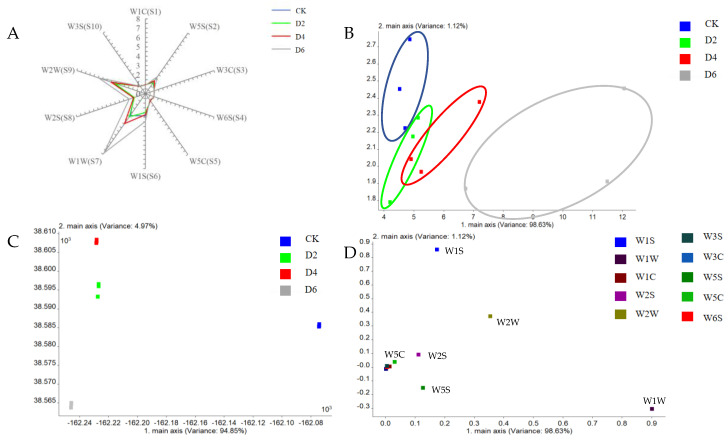
E—nose data of tea at different fermentation times: (**A**) Radar plot (**B**) PCA plot (**C**) LDA plot (**D**) Loading analysis plot.

The radar plot effectively visualizes the response degree of 10 sensors in the electronic nose to the aroma of tea leaves at different fermentation times. Although there are similarities among the sample profiles, variations in signal intensity were observed among certain sensors, particularly W1W, W2W, W1S, W2S, and W5S. This observation showed a significant increase in organic volatiles such as sulfides, organic sulfides, methyl compounds, alcohols, aldehydes, ketones, and nitrogen and oxygen compounds after fermentation. Notably, the most substantial changes occurred after 6 days of fermentation (Figure 2A).

Principal component analysis (PCA) is a comprehensive evaluation method that employs dimensionality reduction calculations to condense multiple variables into a few principal components while maximizing the retention of original index information. According to the results of the PCA analysis, two principal components (PCs), namely PC1 and PC2, were derived, explaining 98.63% and 1.12% of the total variance, respectively, with a cumulative contribution rate of 99.75%, which indicated that these two principal components effectively captured the main aroma information of tea leaves at different fermentation times. The distribution areas of CK, D2, and D4 were adjacent to each other; however, the distribution area of fermented tea for 6 days was far removed from the other samples. Therefore, with the increase in fermentation time, the aroma of tea showed a regular change, and the change in tea aroma was more obvious on the 6th day of fermentation (Figure 2B).

LDA analysis effectively discriminates between groups of samples by preserving all sensor information, thereby bringing similar samples closer together and separating those with larger differences. When applied to the electronic nose data of tea leaves with varying fermentation times, the two principal components account for a total contribution rate of 99.82%, capturing most of the tea aroma information. The distinct distribution patterns observed among the different fermentation time samples indicate significant variations in their aromas. Specifically, D2, D4, and D6 were clustered together on one side, suggesting relatively minor differences among them. However, D2 and D4 exhibit closer proximity to each other, indicating higher similarities in their aroma components (Figure 2C).

Loading analysis can be employed to assess the contribution of the electronic nose sensor in differentiating samples based on their distance from the origin; a greater sensor contribution to the principal component and sample differentiation was observed as it moved further away from the origin. Sensor W1S exhibited the highest displacement in the ordinate direction, while W1W exhibited the greatest displacement in the abscissa direction, indicating that methyl analogs and sulfides significantly contribute to distinguishing tea leaves with varying fermentation times. Additionally, organic sulfides, nitrogen oxides, alcohols, aldehydes, and ketones also play a role in this discrimination. The loading analysis results were consistent with those obtained depicting electronic nose ray response data (Figure 2D).

#### 3.1.2. Volatile Compounds Identified by HS-SPME-GC-MS

**Figure 3:**
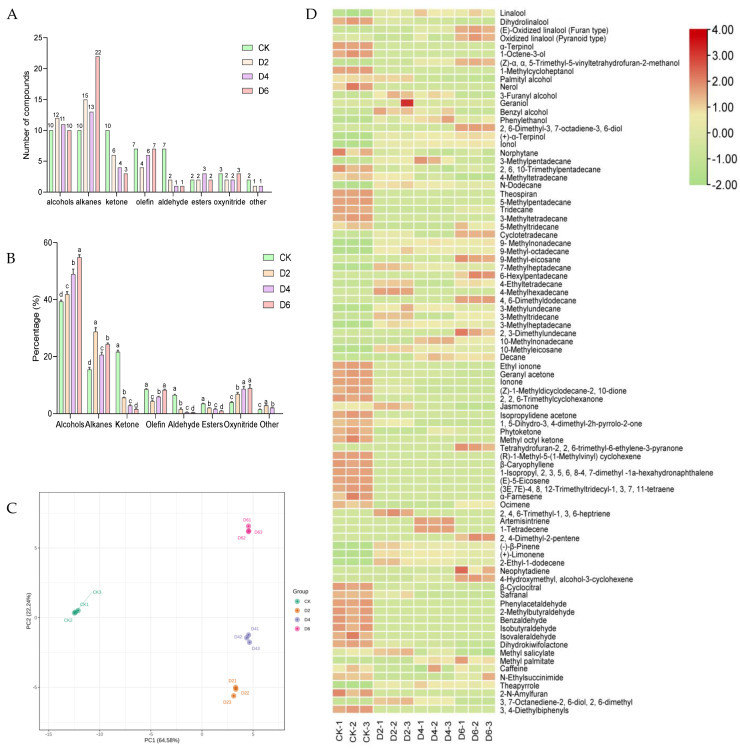
Effect of fermentation time on volatiles in tea (**A**) Amount of different volatile compounds during fermentation (**B**) Proportion of different volatile compounds during fermentation (**C**) PCA plot of volatiles during fermentation (**D**) Thermogram of relative. a, b, c, d different lowercase letters indicate significant differences between groups (*p* < 0.05).

To investigate the distinctive flavor profile of fermented tea at various fermentation durations, we conducted tea extraction to obtain volatile compounds, which were subsequently analyzed using GC-MS. A total of 86 different types of compounds were detected, including 26 alkanes, 16 alkenes, 17 alcohols, 7 aldehydes, 11 ketones, 3 esters, 3 nitrogen oxides, and 3 other substances, were identified. The CK tea leaves, fermented for 2 days, 4 days, and 6 days, respectively, contained a total of 50, 44, 41, and 48 volatile compounds (Figure 3A, Table 1).

The proportion of volatile substances in tea leaves increased with the fermentation time, with alcohol compounds being the most abundant. The content of alcohol compounds in CK, D2, D4, and 6D accounted for 39.25%, 41.80%, 48.88%, and 54.80%, respectively. Alcohol compounds gradually became the main volatile substance during a fermentation period of up to 6 days. Ketones and alkanes were found in CK, accounting for the relative contents of 21.65% and 13.71%, respectively. The content of alkanes and nitrogen oxides progressively increased with fermentation time, reaching 24.32% and 8.95%, respectively, on the sixth day of fermentation (Figure 3B).

Additionally, we conducted a principal component analysis on these 86 compounds. Two principal components (PCs) were obtained from the PCA analysis, namely PC1 and PC2. They accounted for 64.85% and 22.24% of the total variance, respectively. The samples in the CK group exhibited a negative distribution along the PC1 direction, indicating dynamic changes in the relevant products at D2 (Figure 3C).

Alcohol, predominantly characterized by floral and fruity aromas, constitutes the primary aroma compound in most teas. During fermentation, linalool and its derivatives emerged as the dominant alcohol, and the linalool content peaked at D4 relative to CK, showing a significant increase of 35.16% (*p* < 0.05). The pyranic and furan oxidation of linalool exhibited a gradual increment over time during fermentation, demonstrating a significant rise of 70.71% and 225.12%, respectively, compared to CK (*p* < 0.05). However, the relative content of dihydrolinalool gradually declined throughout the fermentation process, with a decrease of 77.14% after 6 days of fermentation (*p* < 0.05).

The substances that underwent the most significant changes during fermentation were (Z)-α,α,5-trimethyl-5-vinyltetrahydrofuran-2-methanol. Its content gradually increased throughout the process, starting at 1.62% in CK and rising to 5.32% after 6 days of fermentation (*p* < 0.05). Post-fermentation analysis did not detect substances such as 1-octene-3-ol, 1-methylcycloheptanol, α-terpinol, however, (+)-α-terpinol, ionol, and phenylethanol were all detectable during the fermentation process. The gradual increase in the (+)-α-terpineol content during fermentation could be attributed to the transformation of α-terpineol into its isomer (+)-α-terpineol. After 6 days of fermentation, the content of (+)-α-terpineol reached 3.13% (*p* < 0.05). Ionol content increased to 3.38% (*p* < 0.05), while the phenylethanol content peaked at 8.05% (*p* < 0.05) and decreased to 4.77% after 6 days of fermentation (*p* < 0.05).

The relative content of dodecane exhibited the highest proportion among alkanes, reaching a maximum value of 6.72% after 2 days of fermentation, which signified a remarkable increase of 460% compared to CK (*p* < 0.05). During fermentation, the content of norphytane, 2,6,10-trimethylpentadecane, and 4-methyltetradecane exhibited a decreasing trend. Specifically, after 6 days, compared to the control group (CK), their content was significantly reduced by 70.64%, 68.60%, and 38.85%, respectively (*p* < 0.05). 3-methylundecane, 9-methylnonadecane, 7-methylheptadecane, and 10-methylnonadecane were newly formed substances during the fermentation process with a relatively high content. On the sixth day of fermentation, their respective contents were 1.33%, 1.22%, 0.89%, and 0.68%.

The content of alkenes was higher in the control group (CK). Specifically, the alkenes present at a higher content in CK tea included (R)-1-methyl-5-(1-methylethenyl) cyclohexene, β-caryophyllene, α-farnesene, 1-isopropyl, 2,3, 5,6,8-4,7-dimethyl -1a-hexahydronaphthalene, and (E)-5-eicosene with a contents of 2.09%, 1.5%, 1.07%, 1.40%, and 1.00%, respectively; these compounds were not detected in teas fermented for D2 or for longer. However, a set of new volatile compounds, including 2-ethyl-1-dodecene, (+)-limonene, and (-)-β-pinene were detected during the fermentation process.

The content of ketones in CK tea was higher in ethyl ionone, geranyl acetone, ionone, and (Z)-1-methylbicyclodecane-2, 10-dione. However, the content decreased as fermentation time increased. Specifically, the content of ethyl ionone gradually decreased with longer fermentation times and decreased by 88.88% (*p* < 0.05) after 6 days of fermentation. Geranyl acetone, ionone, and (Z)-1-methylbicyclodecane-2, 10-dione were not detected after 6 days of fermentation.

The main ester volatiles in tea were dihydrokiwifolactone, and their content decreased as fermentation time increased. CK had 2.62% dihydrokiwifolactone, which could not be detected after 6 days of fermentation. The main aldehydes, β-cyclocitral and safranal, gradually decreased in content during fermentation. At 6 days of fermentation, β-cyclocitral decreased by 82.17% *(p* < 0.05), while safranal was undetectable at 4 days of fermentation. The main nitrogen oxide compound found in tea was theapyrrole, with CK tea exhibiting a low content of only 1.11%. However, after 4 days of fermentation, the theapyrrole content significantly increased to a maximum value of 7.81% (*p* < 0.05), representing a remarkable increase of 603.60%.

### 3.2. Changes of Non-Volatile Components in Light Fermentation Tea

To further investigate the impact of *A. niger* RAF106 fermentation on the nonvolatile compounds present in SGT, we conducted a comprehensive analysis to observe the dynamic changes occurring throughout the fermentation process (Table 2).

The content of non-ester catechin and gallic acid (GA) gradually increased during fermentation, while the content of ester catechin decreased progressively. The (-)-epigallocatechin (EGC) content exhibited a time-dependent increase, reaching its peak after 6 days of fermentation with a significant increase of 123.29% (*p* < 0.05) compared to the control group (CK). Similarly, (-)-epicatechin (EC) content showed an initial rise followed by a decline during fermentation, reaching its maximum value after 4 days with a significant increase of 133.53% (*p* < 0.05) compared to CK. The GA content displayed a gradual increase and reached an astounding increment of 3775% (*p* < 0.05) after 6 days of fermentation. On the other hand, both the (-)-epigallocatechin gallate (EGCG) and (-)-epicatechin gallate (ECG) contents decreased gradually as fermentation progressed, with reductions of 83.25% and 79.39%, respectively (*p* < 0.05), observed after 6 days.

The content of lactic acid and acetic acid was significantly increased in *A. niger* RAF106 fermented tea, establishing them as predominant organic acids following fermentation (*p* < 0.05). Compared to CK, there was a significant increase in the content of lactic acid, acetic acid, and citric acid after fermentation. Lactic acid content reached its peak at 4 days of fermentation with a remarkable increase of 524.58% (*p* < 0.05), while the acetic acid content peaked at 2 days of fermentation with an increase of 411.41% (*p* < 0.05). Additionally, citric acid content increased by 544.39% (*p* < 0.05) at 6 days of fermentation. The content of oxalic acid and fumaric acid in tea exhibited fluctuations during fermentation, with a decrease of 36.82% and 45.98%, respectively, at day 6 (*p* < 0.05). Gradual reductions were observed in the content of quinic acid and malic acid throughout the fermentation process, with a decrease of 30.90% (*p* < 0.05) in quinic acid after 6 days of fermentation and a reduction of 72.20% (*p* < 0.05) in malic acid after the same duration.

The total polyphenol content remained stable throughout the fermentation process. The free amino acid content exhibited a significant increase with a prolonged fermentation time, reaching its peak after 6 days of fermentation and showing an 18.09% increase (*p* < 0.05) compared to the control group (CK). The caffeine content did not show any significant changes during the initial 4 days of fermentation but decreased by 7.46% (*p* < 0.05) on the 6th day compared to CK. The soluble sugar and total flavonoid contents initially increased and then decreased as fermentation progressed, with their highest values observed at 4 days of fermentation, exhibiting a remarkable increase of 95.59% and 54.70%, respectively, compared to CK (*p* < 0.05).

### 3.3. Sensory Evaluation

Based on the color, aroma, and taste of tea, a sensory evaluation was conducted for tea at different fermentation times. Although no statistically significant difference was observed, the sensory evaluation scores of teas fermented by *A. niger* RAF106 exhibited an improvement. The color gradually brightened with prolonged fermentation time. The aroma of the tea intensified with prolonged fermentation, exhibiting a sweet fragrance that aligned with the presence of floral and fruity volatile nitrogen oxides and alcohol in the tea. With an extended fermentation time, the taste gradually evolved into a mellower and sweeter aftertaste accompanied by a balanced level of acidity. This transformation could be attributed to a significant reduction in ester-type catechins, coupled with an increase in the content of free amino acids and soluble sugars (Table 3).

## 4. Discussion

In general, the content of volatile compounds was found to be higher after fermentation compared to before, and the aroma characteristics exhibited a greater intensity post-fermentation. The content of alcohol, alkanes, and nitrogen oxides in tea showed an increase following fermentation, with the alcohol content reaching 54.80% after a 6-day fermentation period (Figure 3A). Linalool and oxidized linalool are among the most prominent aromatic compounds in tea, imparting a floral fragrance (Figure 3D, Table 1) [18]. The tea in question bear resemblance to renowned Chinese spring teas, such as Keemun black tea and Xihu Longjing tea [19,20]. The alkane compound n-dodecane exhibited significant alterations in its content when subjected to the influence of *A. niger* RAF106 (Figure 3D, Table 1). This characteristic facilitates the generation of a distinct *Lonicera japonica* aroma in tea and enhances its freshness following fermentation [21]. The predominant nitrogen–oxygen compounds found in fermented tea are known as tea pyrroles, which contribute to the roasted aroma and nutty undertones in the tea’s aromatic profile. Elevating the content of tea pyrroles enhances fragrance characteristic of fermented tea (Figure 3D, Table 1) [22].

Catechin, a crucial component responsible for the bitterness and astringency found in tea, is also an essential bioactive compound. The fermentation of *A. niger* RAF106 results in a reduction in the content of ester-type catechins while also promoting an increase in the content of non-ester-type catechins and gallic acid (Table 2). During the process of fermentation, microorganisms demonstrate growth and metabolic activities that result in the release of various hydrolytic enzymes such as cellulase, amylase, tannase, and protease, among others. These enzymes aid in breaking down polyphenolic compounds present in tea into smaller molecular entities. As a consequence, ester-type catechins undergo hydrolysis to produce non-ester-type catechins and gallic acid [23,24].

Tea flavor quality greatly relies on the presence of organic acids, which also serve as crucial building blocks or transitional compounds in the development of various flavor components and aromatic substances. These factors significantly contribute to overall tea quality [25]. Following the fermentation of *A. niger* RAF106, the content of lactic acid, acetic acid, and citric acid in tea exhibited a significant increase, with acetic acid and lactic acid being the most predominant acids (Table 2). These findings align with those observed in black tea following Ottoman fermentation [26].

Tea quality can be assessed by examining various factors such as tea polyphenols, total flavonoids, caffeine, free amino acids, and soluble sugars. Among these factors, tea polyphenols and caffeine are known for their contribution to the bitter and astringent taste of tea. Additionally, flavonoids play a significant role in determining the color and flavor characteristics of tea infusion [27]. Free amino acids are primary contributors to the freshness and aroma of tea broth, while soluble sugars serve as the main source of sweetness in tea broth [28,29].

The fermentation of *A. niger* RAF106 did not have a significant impact on tea polyphenols. There was a slight variation in the caffeine content during the initial stages of fermentation, followed by a decrease observed on the sixth day (Table 2). Despite caffeine’s chemical stability, it can be converted into theobromine or other theophyllines through microbial enzymatic activity and under hot and humid environmental conditions. Additionally, caffeine can form compounds with polyphenols and acids to generate salts, resulting in a reduction in the caffeine content after fermentation [30,31]. The total flavonoid content can be significantly enhanced within a short fermentation period by *A. niger* RAF106, with the peak level achieved on the fourth day of fermentation (Table 2). This phenomenon could be attributed to the capacity of *A. niger* to enhance β-glucosidase activity, thereby promoting specific pathways for flavonoid conversion [32]. The free amino acid content gradually increased during fermentation (Table 2), potentially attributed to the degradation or polymerization of amino acids resulting from protein catabolism, hydrolysis, or cleavage in hot and humid environments as well as the enzymatic reactions induced by microbial activity [33,34]. *A. niger* RAF106 fermentation promoted the accumulation of soluble sugars in tea leaves, with the highest content observed at 4 days of fermentation (Table 2). Microbial metabolism during tea processing hydrolyzes or decomposes polysaccharides into soluble sugars. Therefore, the total sugar content can be increased.

Sensory evaluation is a scientific approach widely employed in the food industry for the analysis of food attributes based on human perception, encompassing taste, visual appearance, aroma, and texture. The prolonged fermentation of *A. niger* RAF106 enhanced the overall sensory evaluation scores (Table 3). Volatile and non-volatile substances were enhanced to a certain extent following fermentation, while the interplay between aroma and taste also influenced the sensory perception of flavor [35]. The fermentation of *A. niger* RAF106 potentially enhanced tea quality by facilitating an interaction between the aroma and taste of SGT, thereby augmenting the sensory evaluation of tea to a certain extent.

## 5. Conclusions

The utilization of *A. niger* RAF106 solid-state fermentation for low-value SGT effectively reduced the ester catechin content, significantly increasing the non-ester catechin content in fermented tea and also enhancing the free amino acid content in tea. The fermented tea exhibited an enhanced floral flavor due to the increased levels of linalool, (E)-linalool oxide (furan type), linalool oxide (pyran type), theapyrrole, and (Z)-α,α,5-trimethyl-5-vinyltetrahydrofuran-2-methanol. The resultant fermented tea exhibited a fragrant, mellow, and sweet aftertaste and moderate acidity. Moreover, it effectively enhanced the flavor quality of SGT, thereby laying a foundation for the development of functional tea beverages.

However, this study conducted a preliminary investigation on the fermentation technology of tea, focused on the transformation of endogenous components in tea, specifically examining catechins, free amino acids, caffeine, and organic acids. It is also important to note that there are numerous other components present in tea, such as chlorophyll, flavanols, flavanones, and other flavonoids, which require further investigation regarding their changes, during the fermentation process. This slightly fermented tea differs significantly from the six traditional teas. Due to substantial changes in endoplasmic components, such as polyphenol flavonoids during biotransformation through fermentation, its functional properties can also undergo significant alterations. Therefore, subsequent studies can explore the biological activity of this fermented tea rich in non-ester catechins and use animal models like nematodes and mice to investigate the bioavailability and bioactivity of slightly fermented tea.

## Figures and Tables

**Table 1:** Comparison of volatile substances during the fermentation process.

No.	Volatile Compounds	Relative Content (%)				Odor Description
		CK	D2	D4	D6	
Alcohol						
1	Linalool	10.61 ± 0.29 c	13.28 ± 0.19 b	14.34 ± 0.37 a	13.95 ± 0.55 ab	Floral, sweet ^1^
2	Dihydrolinalool	10.41 ± 0.42 a	4.65 ± 0.22 b	3.24 ± 0.09 c	2.38 ± 0.11 d	Rose, wood, fruity ^2^
3	(E)-Oxidized linalool (Furan type)	4.1 ± 0.12 d	4.98 ± 0.27 c	7.87 ± 0.11 b	13.33 ± 0.39 a	Floral ^1,3^
4	Oxidized linalool (Pyranoid type)	3.79 ± 0.07 b	2.73 ± 0.1 c	3.32 ± 0.88 bc	6.47 ± 0.27 a	Floral ^1,3^
5	α-Terpinol	3.08 ± 0.03	ND	ND	ND	-
6	1-Octene-3-ol	2.76 ± 0.19	ND	ND	ND	Mushroom flavor ^1^
7	(Z)-α,α,5-Trimethyl-5-vinyltetrahydrofuran-2-methanol	1.61 ± 0.01 d	2.41 ± 0.15 c	3.72 ± 0.1 b	5.32 ± 0.09 a	Floral ^2^
8	1-Methylcycloheptanol	1.17 ± 0.02	ND	ND	ND	-
9	Palmityl alcohol	0.98 ± 0.01 b	1.01 ± 0.01 a	0.47 ± 0.02 c	0.41 ± 0.03 d	Floral, oily ^2^
10	Nerol	0.78 ± 0.18	ND	ND	ND	Floral, fresh, citrus ^1^
11	3-Furanyl alcohol	ND	0.79 ± 0.12 a	0.64 ± 0.1 a	ND	-
12	Geraniol	ND	0.26 ± 0.37	ND	ND	Rose ^1,3^
13	Benzyl alcohol	ND	2.2 ± 0.24 a	1.34 ± 0.49 a	ND	Floral ^1,3^
14	Phenylethanol	ND	4.45 ± 1.4 b	8.05 ± 1.06 a	4.77 ± 0.99 b	Rose-like ^1^
15	2,6-Dimethyl-3,7-octadiene-3,6-diol	ND	ND	ND	1.66 ± 0.03	-
16	(+)-α-Terpinol	ND	2.19 ± 0.14 c	2.8 ± 0.03 b	3.13 ± 0.08 a	Floral ^2^
17	Ionol	ND	2.84 ± 0.09 c	3.1 ± 0.22 b	3.38 ± 0.05 a	Violet-like ^2^
Alkanes						-
1	Norphytane	3.61 ± 0.59 a	1.61 ± 0.35 b	0.77 ± 0.32 b	1.06 ± 0.38 b	Alkane-like ^1^
2	3-Methylpentadecane	2.88 ± 0.07 c	4.39 ± 0.09 a	5.29 ± 0.51 a	3.18 ± 0.12 b	-
3	2,6,10-Trimethylpentadecane	1.72 ± 0.15 a	0.58 ± 0.14 b	0.29 ± 0.23 b	0.54 ± 0.13 b	Alkane-like ^1^
4	4-Methyltetradecane	1.39 ± 0.03 a	1.3 ± 0.01 b	1.03 ± 0.04 c	0.85 ± 0.01 d	Alkane-like ^1^
5	N-Dodecane	1.2 ± 0.05 d	6.72 ± 0.22 a	5.94 ± 0.13 b	5.04 ± 0.12 c	Honeysuckle-like ^1^
6	Theospiran	1.17 ± 0.02	ND	ND	ND	Pine wood-like ^2^
7	5-Methylpentadecane	1.08 ± 0.01	ND	ND	ND	Alkane-like ^1^
8	Tridecane	0.93 ± 0.03 a	ND	ND	0.35 ± 0.02 b	Alkane-like ^1^
9	3-Methyltetradecane	0.89 ± 0.04	ND	ND	ND	Alkane-like ^1^
10	5-Methyltridecane	0.57 ± 0.04 a	ND	ND	0.46 ± 0.11 a	-
11	Cyclotetradecane	ND	0.46 ± 0.02 a	ND	0.91 ± 0.01 a	-
12	9-Methylnonadecane	ND	1.09 ± 0.02 b	1.31 ± 0.05 a	1.22 ± 0.09 ab	Alkane-like ^1^
13	9-Methyl-octadecane	ND	0.55 ± 0.05 a	0.47 ± 0.02 a	0.48 ± 0 a	Alkane-like ^1^
14	9-Methyl-eicosane	ND	ND	ND	0.5 ± 0.04	-
15	7-Methylheptadecane	ND	1.65 ± 0.08 a	1.13 ± 0.02 b	0.89 ± 0.01 c	-
16	6-Hexylpentadecane	ND	ND	ND	0.36 ± 0.07	-
17	4-Ethyltetradecane	ND	0.59 ± 0.01 a	ND	0.5 ± 0.02 b	-
18	4-Methylhexadecane	ND	0.72 ± 0.01 a	ND	ND	-
19	4,6-Dimethyldodecane	ND	ND	ND	0.5 ± 0.01	-
20	3-Methylundecane	ND	2.07 ± 0.36 a	1.88 ± 0.08 a	1.33 ± 0.05 b	-
21	3-Methyltridecane	ND	5.9 ± 0.09 a	ND	4.14 ± 0.08 a	Alkane-like ^1^
22	3-Methylheptadecane	ND	0.41 ± 0.03 a	0.38 ± 0.02 a	0.31 ± 0.03 b	Alkane-like ^1^
23	2,3-Dimethylundecane	ND	ND	ND	0.22 ± 0.04	-
24	10-Methylnonadecane	ND	ND	1.13 ± 0.06 a	0.68 ± 0.02 b	-
25	10-Methyleicosane	ND	0.64 ± 0 a	0.51 ± 0.02 b	0.4 ± 0.01 c	-
26	Decane	ND	ND	0.44 ± 0.04 a	0.39 ± 0.01 a	-
ketone						
1	Ethyl ionone	8.99 ± 0.12 a	2.3 ± 0.06 b	1.69 ± 0.01 c	1 ± 0.07 d	Violet-like ^2^
2	Geranyl acetone	4.02 ± 0.13 a	0.44 ± 0.05 c	0.6 ± 0 b	ND	Fruital ^2^
3	Ionone	2.11 ± 0.01 a	0.36 ± 0 b	ND	ND	Floral, violet ^1,3^
4	(Z)-1-Methyldicyclodecane-2,10-dione	1.92 ± 0.09 a	0.7 ± 0.03 b	ND	ND	-
5	2,2,6-Trimethylcyclohexanone	1.19 ± 0.05	ND	ND	ND	-
6	Jasmonone	0.93 ± 0.04 b	1.37 ± 0.09 a	0.31 ± 0.02 c	ND	Floral ^2^
7	Isopropylidene acetone	0.8 ± 0.03	ND	ND	ND	Mint, honey-like ^2^
8	1,5-Dihydro-3,4-dimethyl-2h-pyrrolo-2-one	0.69 ± 0.02 a	0.45 ± 0.04 b	ND	ND	White bread crust ^2^
9	Phytoketone	0.63 ± 0.04 a	ND	0.22 ± 0.16 b	0.16 ± 0.01 b	Oily, woody ^2^
10	Methyl octyl ketone	0.32 ± 0.04	ND	ND	ND	Floral ^2^
11	Tetrahydrofuran-2,2,6-trimethyl-6-ethylene-3-pyranone	ND	ND	ND	0.42 ± 0.04	Floral, honey-like ^2^
Olefins						
1	(R)-1-Methyl-5-(1-Methylvinyl) cyclohexene	2.09 ± 0.02	ND	ND	ND	-
2	β-Caryophyllene	1.5 ± 0.05	ND	ND	ND	Floral ^1^
3	1-Isopropyl, 2,3, 5,6,8-4,7-dimethyl -1a-hexahydronaphthalene	1.4 ± 0.01	ND	ND	ND	Herbal, woody ^2^
4	(E)-5-Eicosene	1 ± 0.01	ND	ND	ND	-
5	(3E,7E)-4,8,12-Trimethyltridecyl-1,3, 7,11-tetraene	0.88 ± 0.03	ND	ND	ND	-
6	α-Farnesene	1.07 ± 0.19	ND	ND	ND	Citrus, herbal ^2^
7	Ocimene	0.58 ± 0.04 a	ND	ND	0.42 ± 0.01 b	Citrus, sweet ^2^
8	Artemisintriene	ND	ND	0.52 ± 0.02	ND	-
9	2,4,6-Trimethyl-1,3,6-heptriene	ND	0.73 ± 0.06	ND	ND	-
10	1-Tetradecene	ND	ND	1.04 ± 0.01	ND	-
11	2,4-Dimethyl-2-pentene	ND	ND	ND	0.12 ± 0.02	-
12	(-)-β-Pinene	ND	0.5 ± 0.03 a	0.38 ± 0.02 b	0.35 ± 0.03 b	-
13	(+)-Limonene	ND	1.39 ± 0.14 a	1.39 ± 0.02 a	1.09 ± 0.03 b	Fruity, lemon-like ^2^
14	2-Ethyl-1-dodecene	ND	1.7 + 0.15 a	1.26 + 0.04 b	0.96 + 0.04 c	-
15	Neophytadiene	ND	ND	ND	0.13 + 0.04	-
16	4-Hydroxymethyl, alcohol-3-cyclohexene	ND	ND	1.3 ± 0.18 b	5.22 ± 0.26 a	-
Aldehydes						
1	β-Cyclocitral	2.3 ± 0.06 c	0.67 ± 0.12 a	0.54 ± 0.03 a	0.41 ± 0.02 b	Fruity ^2^
2	Safranal	1.48 ± 0.03 a	0.85 ± 0.27 b	ND	ND	Woody, spicy, phenolic ^1^
3	Phenylacetaldehyde	0.87 ± 0.03	ND	ND	ND	Honey ^1^
4	2-Methylbutyraldehyde	0.78 ± 0.04	ND	ND	ND	Fruity ^1^
5	Benzaldehyde	0.6 ± 0.03	ND	ND	ND	Caramel, fruity, bitter almond, burnt sugar ^1^
6	Isobutyraldehyde	0.28 ± 0.03	ND	ND	ND	-
7	Isovaleraldehyde	0.23 ± 0.04	ND	ND	ND	-
Esters						
1	Dihydrokiwifolactone	2.61 ± 0.05 a	0.91 ± 0.04 b	0.49 ± 0.02 c	ND	Sweet ^2^
2	Methyl salicylate	0.87 ± 0.07 b	1.11 ± 0.03 a	0.76 ± 0.11 b	0.52 ± 0.05 c	Peppermint ^1,3^
3	Methyl palmitate	ND	ND	0.37 ± 0.02 a	0.43 ± 0.1 a	Oily, waxy, fatty ^1^
Oxynitride						
1	Caffeine	1.62 ± 0.21 a	0.55 ± 0 b	0.84 ± 0.95 ab	1.19 ± 0.31 a	-
2	N-Ethylsuccinimide	1.21 ± 0.03 a	ND	ND	0.86 ± 0.39 a	-
3	Theapyrrole	1.11 ± 0.02 c	6.27 ± 0.48 b	7.81 ± 0.42 a	6.9 ± 0.52 ab	-
Other						
1	2-N-Amylfuran	0.88 ± 0.13	ND	ND	ND	-
2	3,7-Octanediene-2,6-diol, 2,6-dimethyl	ND	2.89 ± 0.13 a	2.07 ± 0.05 b	ND	-
3	3,4-Diethylbiphenyls	0.58 ± 0.03	ND	ND	ND	-

a, b, c, d different lowercase letters indicate significant differences between groups (*p* < 0.05); ND, not detectable. Odor description was obtained from: ^1^ the literature with a database (Flavornet: The LRI and Odour Database); ^2^ https://baike.baidu.com/, accessed on 10 August 2023; ^3^ https://www.favornet.org/favornet.html, accessed on 10 August 2023; “-”not defined.

**Table 2:** Changes of non-volatile components during fermentation.

Compound	Content (%)			
	CK	D2	D4	D6
Catechins and gallic acid *				
EGC	1.46 ± 0.089 c	2.79 ± 0.153 b	3.15 ± 0.239 a	3.26 ± 0.101 a
C	0.45 ± 0.027 a	0.52 ± 0.031 a	0.45 ± 0.008 a	0.41 ± 0.062 a
EC	1.67 ± 0.092 b	3.96 ± 0.143 a	4.09 ± 0.196 a	3.90 ± 0.18 a
Non-ester catechins	3.58 ± 0.123 c	7.27 ± 0.181 b	7.69 ± 0.437 a	7.57 ± 0.161 ab
EGCG	3.88 ± 0.236 a	1.32 ± 0.08 b	0.89 ± 0.025 c	0.65 ± 0.034 c
ECG	4.27 ± 0.208 a	1.86 ± 0.103 b	1.13 ± 0.044 c	0.88 ± 0.006 c
Ester catechins	8.15 ± 0.441 a	3.17 ± 0.181 b	2.02 ± 0.066 c	1.53 ± 0.033 d
GA	0.12 ± 0.003 c	3.35 ± 0.086 b	4.30 ± 0.218 a	4.65 ± 0.139 a
Organic acid				
Oxalic acid	1.04 ± 0.074 a	0.42 ± 0.097 c	0.87 ± 0.161 ab	0.66 ± 0.055 bc
Quinic acid	3.73 ± 0.272 a	3.14 ± 0.299 b	2.22 ± 0.103 d	2.57 ± 0.154 c
Malic acid	4.04 ± 0.394 a	3.03 ± 0.201 b	1.74 ± 0.074 c	1.12 ± 0.086 d
Lactic acid	4.63 ± 0.71 c	24.83 ± 4.184 b	28.89 ± 1.083 a	24.1 ± 3.075 b
Acetic acid	3.13 ± 0.476 d	16 ± 0.991 a	13.05 ± 1.316 b	9.48 ± 1.532 c
Fumaric acid	0.36 ± 0.043 b	0.34 ± 0.061 b	0.5 ± 0.033 a	0.2 ± 0.017 c
Citric acid	0.21 ± 0.017 c	0.23 ± 0.03 c	0.86 ± 0.064 b	1.38 ± 0.073 a
Others				
Tea polyphenols	18.34 ± 0.33 a	20.59 ± 0.644 a	19.66 ± 1.032 a	20 ± 0.692 a
Amino acid	4.73 ± 0.138 c	5.11 ± 0.181 bc	5.18 ± 0.071 ab	5.59 ± 0.137 a
Caffeine	3.86 ± 0.015 a	3.82 ± 0.044 a	3.93 ± 0.023 a	3.57 ± 0.023 b
Soluble sugar	0.68 ± 0.044 c	1.29 ± 0.085 ab	1.33 ± 0.045 a	1.16 ± 0.01 b
Total flavone	0.75 ± 0.06 c	1.04 ± 0.074 ab	1.16 ± 0.08 a	0.84 ± 0.03 bc

* (+)-catechin (C), (-)-epigallocatechin (EGC), (-)-epicatechin (EC), non-ester catechins (EGC, C and EC), (-)-epicatechin gallate (ECG), (-)-epigallocatechin gallate (EGCG), ester catechins (EGCG and ECG), and gallic acid (GA). a, b, c, d different lowercase letters indicate significant differences between groups (*p* < 0.05).

**Table 3:** Sensory evaluation of tea during fermentation.

Sample	Color ^1^	Aroma ^1^	Taste ^1^	Total Ccore ^2^	General Evaluation ^3^
CK	81.5 ± 4.44	81.63 ± 9.395	88.38 ± 3.377	84.97 ± 4.90	Dull yellow, slightly harsh and stale odour, plain and thin
D2	90.3 ± 4.41	86.83 ± 2.63	87.83 ± 5.07	88.03 ± 2.81	Bright yellow, fragrant, mellow and normal
D4	89.83 ± 4.7	85.33 ± 4.03	89.17 ± 2.78	88.15 ± 2.44	Bright yellow, fragrant, sweet after taste
D6	90.01 ± 5.014	88.63 ± 6.39	92.38 ± 2.875	90.92 ± 2.855	Bright yellow, fragrant, mellow and sweet after taste, moderate acidity

^1^, According to the sensory scoring criteria (Table S3), tea is evaluated based on its color, aroma, and taste, with a maximum score of 100. The higher the score, the more acceptable the tea becomes. ^2^, The total score was calculated by applying Equation (1) with formula 2.6. ^3^, The color, aroma, and taste of tea were comprehensively evaluated after the sensory experience.

## Data Availability

Data are contained within the article.

## Supplementary Materials

The following supporting information can be downloaded at: https://www.mdpi.com/article/10.3390/foods12183420/s1, Table S1. Configuration of the mobile phase; Table S2. Standard curve for each substance; Table S3. Sensory scoring criteria; Figure S1. HPLC chromatogram; Figure S2. Total ion flow diagram of volatile substances during tea fermentation determined by SPME-GC-MS/MS.
