# Supplementary material for: Enhancing the Flavor Profile of Summer Green Tea via Fermentation with Aspergillus niger RAF106
Source: Foods. 2023 Sep 14;12(18):3420. doi: 10.3390/foods12183420 (PMC10529516; doi:10.3390/foods12183420)
Supplement: Supplementary file 1 [file foods-12-03420-s001.zip › Supplementary Figures.pdf]

Supplementary Figures

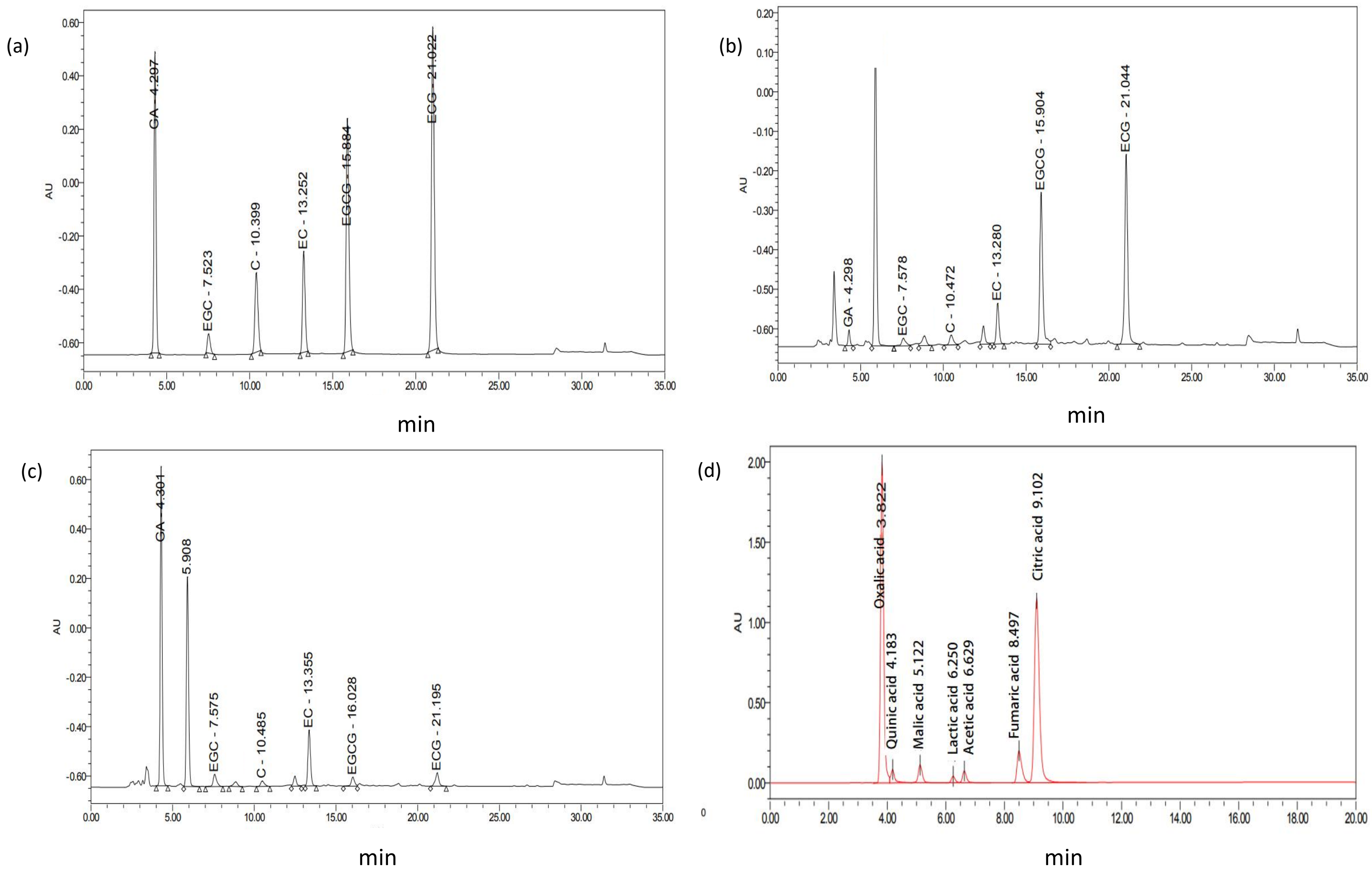

Figure S1. HPLC chromatogram (a) catechin standard (b) catechin CK sample (c) catechin D6 sample (d) organic acid standard sample.

The non-fermented tea and the teas fermented for 2, 4, and 6 days are designated as CK, D2, D4, and D6 respectively.

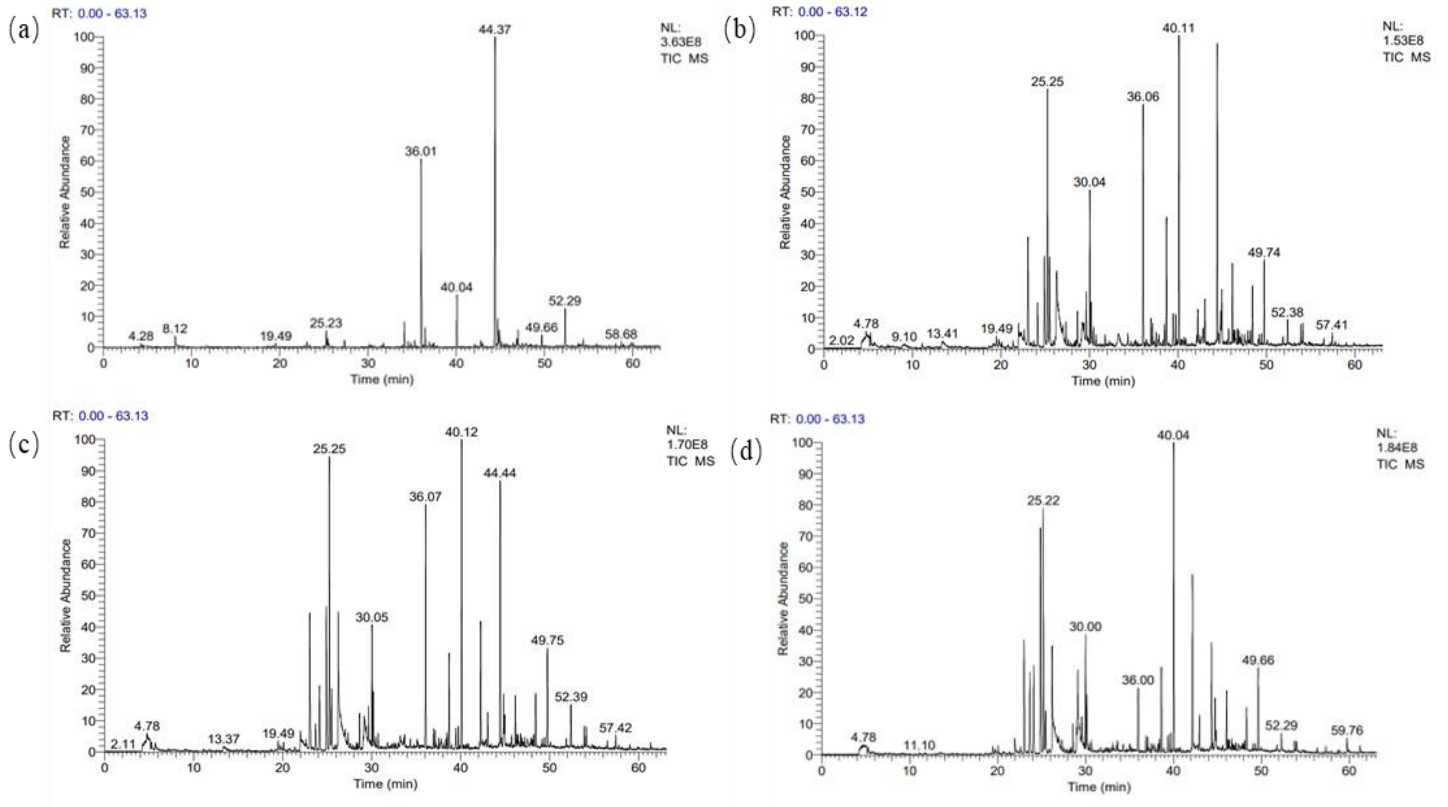

Figure S2. Total ion flow diagram of volatile substances during tea fermentation determined by SPME-GC-MS/MS (a) CK (b) D2 (c) D4 and (d) D6
