# Supplementary material for: Enhancing the Flavor Profile of Summer Green Tea via Fermentation with Aspergillus niger RAF106
Source: Foods. 2023 Sep 14;12(18):3420. doi: 10.3390/foods12183420 (PMC10529516; doi:10.3390/foods12183420)
Supplement: Supplementary file 1 [file foods-12-03420-s001.zip › Supplementary Tables.pdf]

Table S1. Configuration of the mobile phase

Compound	Mobile Phase	
	A	B
Ammonium acetate（g）	3.84	1.542
Acetonitrile (mL)	64	224
Tetrahydrofuran (mL)	16	56
Purified water (mL)	720	120
Trifluoroacetic acid (mL)	3	1.5
pH	3.0	3.0

Table S2. Standard curve for each substance

Substance*	Standard Curve
GA	y = 4916.5x + 4124.7, R^2 = 0.9999
EC	y = 1961.9x - 8526.3, R^2 = 1
C	y = 2091.8x - 13683, R^2 = 1
EGC	y = 583.99x - 4927.7, R^2 = 1
EGCG	y = 6209.2x - 502473, R^2 = 0.9994
ECG	y = 8077.4x - 701884, R^2 = 0.9988
Oxalic acid	y = 976.61x + 114888,R^2 = 0.9997
Malic acid	y = 109.05x - 10986, R^2 = 0.999
Acetic acid	y = 46.262x - 1719.5, R^2 = 1
Citric acid	y = 546.39x - 6793.8, R^2 = 1
Lactic acid	y = 37.222x - 591.6, R^2 = 1
Quinic acid and	y = 81.103x - 8928, R^2 = 0.9992
Fumaric acid	y = 449.34x - 224.78, R^2 = 0.9998

*(+)-catechin (C), (-)-epigallocatechin (EGC), (-)-epicatechin (EC), (-)-epicatechin gallate (ECG), (-)-epigallocatechin gallate (EGCG), and gallic acid (GA)

Table S3. Sensory scoring criteria*

Character	Evaluation	Score
Color	Bright yellow and clear	≥90
	Light yellow	80-89
	Dull yellow	61-79
	Suspension or precipitate	≤60
Aroma	Fragrant and lasting	≥90
	Pure and normal	80-89
	Less pure	61-79
	Harsh odour, stale odour and tainted odour	≤60
Taste	Mellow and sweet after taste, moderate acidity	≥90
	Mellow and normal, sweet after taste, sour taste	80-89
	Plain and thin	61-79
	Slightly astringent, strong sour taste, bitter taste tainted taste	≤60

* The development of this scoring standard primarily relies on referencing Table B8 'Comments on the quality of yellow tea and score sheet for each quality factor' (page 22) in GB/T 23776-2018 by the China National Institute of Standardization (CNIS), along with Section 2 'General Tea' (pages 6-13) in GB/T 14487-2017 'Tea vocabulary for sensory evaluation'.
